# *Lactobacillus rhamnosus* CNCMI-4317 Modulates *Fiaf/Angptl4* in Intestinal Epithelial Cells and Circulating Level in Mice

**DOI:** 10.1371/journal.pone.0138880

**Published:** 2015-10-06

**Authors:** Elsa Jacouton, Núria Mach, Julie Cadiou, Nicolas Lapaque, Karine Clément, Joël Doré, Johan E. T. van Hylckama Vlieg, Tamara Smokvina, Hervé M Blottière

**Affiliations:** 1 Danone Nutricia Research, Palaiseau, France; 2 INRA, UMR 1319 Micalis, Jouy en Josas, France; 3 AgroParistech, UMR Micalis, Jouy en Josas, France; 4 INRA, US 1367, Metagenopolis, Jouy en Josas, France; 5 INSERM, U872, centre de recherche des Cordeliers, Paris, France; 6 UPMC, Paris, France; 7 ICAN, APHP, CNRH-Ile de France, Paris, France; University of Guelph, Canada, CANADA

## Abstract

**Background and Objectives:**

Identification of new targets for metabolic diseases treatment or prevention is required. In this context, FIAF/ANGPTL4 appears as a crucial regulator of energy homeostasis. Lactobacilli are often considered to display beneficial effect for their hosts, acting on different regulatory pathways. The aim of the present work was to study the effect of several lactobacilli strains on *Fiaf* gene expression in human intestinal epithelial cells (IECs) and on mice tissues to decipher the underlying mechanisms.

**Subjects and Methods:**

Nineteen lactobacilli strains have been tested on HT–29 human intestinal epithelial cells for their ability to regulate *Fiaf* gene expression by RT-qPCR. In order to determine regulated pathways, we analysed the whole genome transcriptome of IECs. We then validated *in vivo* bacterial effects using C57BL/6 mono-colonized mice fed with normal chow.

**Results:**

We identified one strain (*Lactobacillus rhamnosus* CNCMI–4317) that modulated *Fiaf* expression in IECs. This regulation relied potentially on bacterial surface-exposed molecules and seemed to be PPAR-γ independent but PPAR-α dependent. Transcriptome functional analysis revealed that multiple pathways including cellular function and maintenance, lymphoid tissue structure and development, as well as lipid metabolism were regulated by this strain. The regulation of immune system and lipid and carbohydrate metabolism was also confirmed by overrepresentation of Gene Ontology terms analysis. *In vivo*, circulating FIAF protein was increased by the strain but this phenomenon was not correlated with modulation *Fiaf* expression in tissues (except a trend in distal small intestine).

**Conclusion:**

We showed that *Lactobacillus rhamnosus* CNCMI–4317 induced *Fiaf* expression in human IECs, and increased circulating FIAF protein level in mice. Moreover, this effect was accompanied by transcriptome modulation of several pathways including immune response and metabolism *in vitro*.

## Introduction

Over the last decades, increased obesity is associated with increased metabolic syndromes characterized by type-2-diabetes (T2D), cardiovascular diseases (CVD) or low-grade inflammation. Regarding the increased prevalence of these diseases, scientific interest has emerged in developing new therapeutic approaches. The recognition of FIAF (Fasting Induced Adipose Factor) protein as a central regulator of energy homeostasis emphasized it as a strong candidate in obesity-associated disorders treatment and/or prevention. FIAF also known as ANGTPL4 (angiopoietin-like 4), is an adipokine expressed in several tissues including adipose tissue, liver, intestine and heart. With increasing studies on FIAF, it seems that its physiological effects are tissues dependent. FIAF inhibits lipoprotein lipase (LPL) and promotes lipolysis resulting in increased triglycerides (TGs) serum level and decreased free fatty acids (FA) and cholesterol uptake into different tissues [1, 2]. Although a direct interaction has been established, exact mechanism of LPL inhibition is still not fully elucidated [3–5].

Conflicting data on the role of FIAF in glucose and lipid metabolism have been reported. Even transgenic mice showed impairment in glucose tolerance, overexpression of *Fiaf* gene in diabetic mice improvesd hyperglycemia and glucose tolerance [1, 6]. *In vivo*, rodent experiments have associated FIAF to hyperlipidemia, caused by decreasing very low-density lipoprotein (VLDL) clearance [7]. These data were supported by human genetic studies, which revealed lower plasma TGs in E40K variant [8, 9]. However, other studies failed to correlate FIAF level to plasma TGs levels [10]. FIAF beneficial effects have been reported against inflammation induced by high fat diet (HFD) by limiting macrophages lipid overload [11] and in no reflow protection after cardiac infarcts [12]. Recently, a higher circulating *Fiaf* level has been described in people characterized by a low gene count (LGC) microbiome and associated with marked inflammatory phenotype and adiposity [13].

Thus, FIAF displays a critical role in lipid and glucose metabolism even if more knowledge about mechanisms of action is required to better understand the physiological effects of FIAF regulation.

*Fiaf* gene is considered as a target gene of peroxisome proliferator-activated receptors (PPARs) but several others regulators including glucocorticoids, and recently biliary acids have been described as *Fiaf* mediators [14–16]. Mice exhibiting a conventional microbiota but with intestinal *Fiaf* gene suppression are not protected against HFD-induced obesity as their GF counterparts [17] showing microbiota driven *Fiaf gene* regulation.

More and more evidences revealed that some probiotics up-regulate intestinal FIAF expression through reactive oxygen species (ROS) or short chain fatty acids (SCFA) release [18, 19]. Recently, a transcriptome analysis of murine jejunum revealed the induction of *Fiaf* after *Lactobacillus rhamnosus* (*L*. *rhamnosus*) HN001 administration [20]. *Lactobacillus paracasei* (*L*. *paracasei*) *F19* induced *Fiaf* gene expression in a PPAR-γ, PPAR-α dependent manner and decreased fat storage under HFD. This effect seemed mediated by a non-identified secreted compound [21]. Thus, molecular mechanisms and microbial effectors regulating its expression are still poorly understood.

Lactobacilli largely used in daily food and especially in fermented dairy products, can be delivered in amount up to 10^12^ live bacteria into the digestive tract. Thus, being in direct contact with the intestinal mucosa, lactobacilli represent a large source of potential regulators of host physiology. In this context, we assessed the ability of 19 bacterial strains of *L*. *paracasei* and *L*. *rhamnosus* species to modulate *Fiaf* gene expression in IECs. In order to dig into the biological mechanism involved, we realized a whole genome transcriptome analysis of epithelial cells in contact with different bacterial strains. Finally, we used mono-colonized mice to validate *Fiaf* regulation in an *in vivo* model and to determine the impact of its modulation on host physiology.

## Material and Methods

### Epithelial cells culture and reagents

The human intestinal epithelial cell lines HT–29 was obtained from the American Type Culture Collection (ATCC, Rockville, MD). HT–29 cells were cultured in DMEM supplemented with 10% heat-inactivated fetal calf serum (FCS), 2 mM L-glutamine (Sigma), 1X Non essential Amino acid (Invitrogen), penicillin (50 IU/ml) and streptomycin (50 μg/ml) in an humidified atmosphere containing 10% CO2 at 37°C. After seeding, cells were grown 48h in 6 or 12 wells plate in antibiotic-free medium at 3.25X10^5^ and 6.5X10^5^ respectively. Medium was changed just before the addition of bacterial or reagents for 6h.

Rosiglitazone (used as positive control), GW9662, GW6471 and GW7647 (Cayman chemicals) were dissolved in DMSO following the manufacturer’s instructions and diluted at 100μM in antibiotic-free DMEM. They were used at a final concentration of 10μM except for GW6471 at 1μM. The antagonists (GW9662, GW6471) were added 1h before challenging with rosiglitazone or GW7647 respectively.

### Bacterial strains culture and screening

Bacteria from Danone collection (Table A in S1 File) were cultivated in MRS (Man, Rogosa and Sharpe medium, Oxoid CM0359) at 37°C in pseudo-aerobic condition. Bacterial cultures (stationary phase) were centrifuged at 5,000x g for 10 min. Conditioned media (CM) were then collected, and filtered on 0.2*μ*m PES filters. Bacterial pellets were washed twice in PBS and resuspended in antibiotic-free DMEM at OD_600_ = 0.1 (corresponding to mean Multiplicity Of Infection ranging from 23 to 113 bacteria for 1 cell) for bacteria. Cells were stimulated with 20% final volume of bacterial culture.

To respect the same ratio (bacteria/cell), Heat Inactivated (HI) bacteria, prepared at OD_600_ = 1 and heated at 80°c for 20 min, were added at 10% of final volume.

Conditionned media (CM), were used at 10% of final volume to limit the presence of lactic acid.

Transwell^TM^ permeable support (Corning) was used to separate bacterial strain from cells for contact dependency test. In those assay, HT–29 were grown in the bottom of 24-well plates, transwell were then added and bacteria were seeded in the transwell preventing direct contact.

### RNA extraction and quantitative real-time PCR (RT-qPCR) of *Fiaf* gene on HT–29 cells

Total RNA of HT–29 cells incubated with different bacterial strains was extracted using Qiashredders column and purified using RNeasy mini-kit (Qiagen, Courtaboeuf, France) according to the manufacturer's recommendations. RNA concentration was measured by using a NanoDrop spectrophotometer (NanoDrop Technologies, Wilmington, USA), and the RNA integrity value (RIN) was assessed by using a 2100 Bioanalyzer (Agilent Technologies Inc., Santa-Clara, USA). All samples had a RIN above 9,6. Briefly, cDNAs synthesis was realized from 1μg of RNA using High Capacity cDNA Reverse Transcription kit (Applied Biosystems, USA) according to the manufacturer’s instructions. cDNAs were diluted at 20ng/ml. RT-qPCR were carried out with Taqman probes (Life technologies, France; Table B in S1 File) according to manufacturer instructions using an ABI Prism 7700 (Applied biosystems, USA) thermal cycler in a reaction volume of 25μl. For each sample and each gene, PCR run were performed in triplicates. In order to quantify and normalize the expression data, we used the ΔΔCt method using the geometric mean Ct value from *β-Actin* and *Gapdh* as the endogenous reference genes [22].

### Microarray analysis of HT–29 cells

Raw microarray data have been deposited in the GEO database under accession no. (GSE62311).

A total of 28 microarrays were analysed: 8 replicates of HT–29 cells at different passage number for *L*. *rhamnosus* CNCMI–4317 treatment, rosiglitazone (positive control) and DMEM (negative control) and four replicates for *L*. *rhamnosus* CNCMI–2493. We used the Illumina human genome microarrays (HumanHT_12 v4 Expression BeadChip Kit, SanDiego,USA). For each sample, 750ng of labelled cDNA was synthesized from total RNA using Ovation PicoSL WTA System v2 and Encore BiotinIL Module kits (Nugen Technologies, Inc. Leek, The Netherlands). The slides were scanned with iScan Illumina and data recovered using GenomeStudio Illumina software (version 1.0.6). All microarray analyses, including pre-processing, normalization and statistical analysis were carried out using 'Bioconductor' packages in R programming language (version 3.0.2) (for more detail concerning data normalization, see S1 file).

The list of differentially expressed (DE) genes were uploaded into using Ingenuity Software (IPA; version 5.5, Ingenuity Systems, Redwood City, CA) to identify relevant molecular functions, cellular components and biological processes using a right-tailed Fisher’s exact test. IPA computed networks and ranked them following a statistical likelihood approach [23]. All networks with a score of 25 and at least 30 focus genes were considered to be biologically relevant.

Additionally, ErmineJ software program was used as a complementary method to relate changes in gene expression to functional changes. ErmineJ software program is based on overrepresentation of Gene Ontology (GO) terms. GO terms were considered significantly at a FDR < 5%.

To technically validate the data generated in the microarray study, quantitative RT-qPCR was carried out on 12 selected candidate genes (Table B in S1 File, see S1 File for more detail). This set of genes were analysed using a linear effect model, including treatment of interest as a fixed effect. Differences were considered significant at *P* <0.05.

### *In vivo* experiment

All experiments were handled in accordance with the institutional ethical guidelines. The “Comité d’Ethique en Expérimentation Animale of the Centre INRA of Jouy-en-Josas and AgroParisTech—COMETHEA” ethics committee approved the study. Seven to eleven weeks-old germ-free (GF) C57BL/6 mice (CNRS-CDTA, Orléans, France) were maintained in sterile isolators at INRA ANAXEM germ-free animal facility, 3 to 5 per cages, on *ad libitum* irradiated normal chow (R 03–40, SAFE) in 12h light cycles. Temperature and moisture were carefully controlled. Mice were observed once a day to ensure their welfare. Mice were separated in three groups depending on bacterial gavage. Mice were colonized by one-time gavage of *L*. *rhamnosus* CNCMI–4317 (n = 7), *L*. *rhamnosus* CNCMI–2493 (n = 6) prepared at 1X10^9^CFU/ml in PBS or PBS as control treatment (n = 5). Body weight was recorded twice a week after bacterial or PBS gavage. After eleven days, mice were sacrificed by cervical dislocation and all tissues (intestine, adipose tissues and liver) were removed and flushed with ice-cold PBS within the next 30 minutes. Tissues were immediately snap frozen in liquid nitrogen and stored at -80°C until processed. Colonization was confirmed by bacterial counting of feces and API 50CH (Biomerieux, France).

### Mouse serum analysis

Lipoproteins (FA, total cholesterol, HDL, LDL and triglycerides) and cytokines dosages (GM-SCF, Il–1β, Il–2, Il–4, Il–5, Il–6, Il–7, Il–10, Il-12p70, TNF-α, MCP–1, IFN-γ) were realized on the Anexplo platform (Toulouse, France) using Pentra 400 instrumentation and Milliplex mouse kit (Millipore) coupled to Luminex technology respectively.

Serum FIAF was determined using Angiopoietin-like protein 4, ANGPTL4, ELISA Kit (MyBiosource, San Diego, USA).

### Statistical analysis of RT-qPCR data and metabolites

All data were normally distributed. The values presented herein are expressed as means ± standard deviation (SD). Data were analysed using one-way ANOVA followed by Turkey multiple post-hoc test Graph Pad Prism (version 5). Differences were considered significant at *P* <0.05. A linear regression was conducted to evaluate the association between RT-qPCR and microarray expression.

## Results

### *Fiaf* is up regulated by *L*. *rhamnosus* CNCMI–4317 strain in IECs

**Fig 1:**
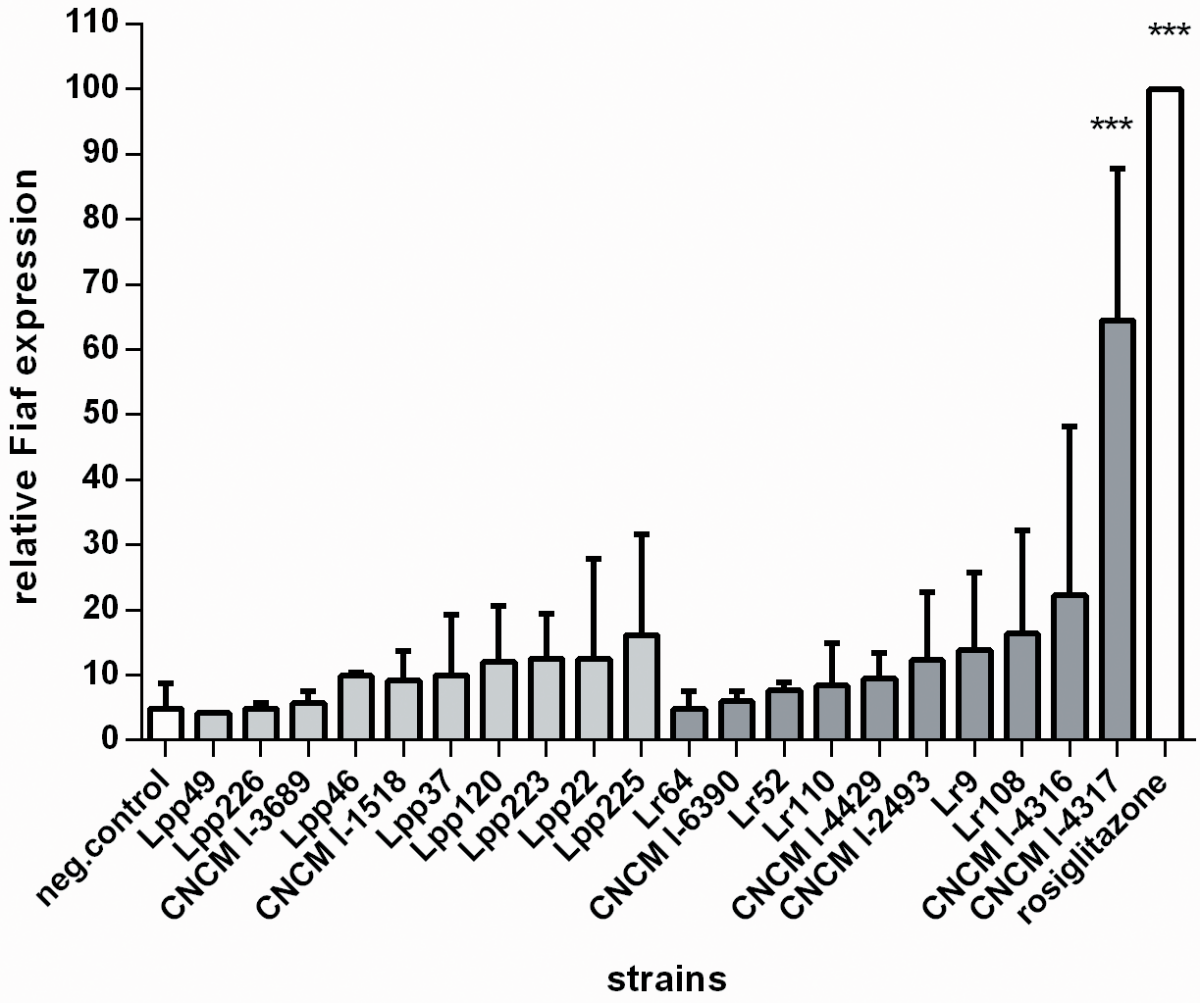
Effect of *L*. *rhamnosus* and *L*. *paracasei* on *Fiaf* expression in IECs. Cells were stimulated 6h with 20% of final volume of bacterial cultures. Bars represent mean of *Fiaf* relative expression (percentage of rosiglitazone) from two to seven independent experiments performed in triplicates. Clear bars correspond to *L*. *paracasei* strains and dark bars correspond to *L*. *rhamnosus* strains. Data are normalized using β-*Actin* as control gene. Stars represent p<0.001 (***) in comparison with negative control (DMEM).

In order to evaluate the potential of the two *Lactobacillus* species (*L*. *rhamnosus*, *L*. *paracasei*) in regulating host metabolism, we tested 19 bacterial strains for their ability to modulate *Fiaf* gene expression in IECs by RT-qPCR. HT–29 human epithelial cells were exposed to each bacteria at OD_600nm_ = 0.1 for 6h before RNA extraction. Among the 10 *L*. *paracasei* and 9 *L*. *rhamnosus* strains tested (detailed in Table A in S1 File), *L*. *rhamnosus* CNCMI–4317 showed the most effective activation of *Fiaf* gene expression (*P*<0.001) (Fig 1) suggesting a strain specific effect. This activation corresponded to about 65% of the one induced by rosiglitazone, a selective PPAR-γ ligand (used as positive control). So, we decided to focus our mechanistic analysis on the bacterial strain using *L*. *rhamnosus* CNCMI–2493 as bacterial negative control and *L*. *Rhamnosus* CNCM–4317.

### *L*. *rhamnosus* CNCMI–4317 strain induced the expression of *Fiaf gene* in a PPAR-γ independent but PPAR-α dependent manner in IECs

**Fig 2:**
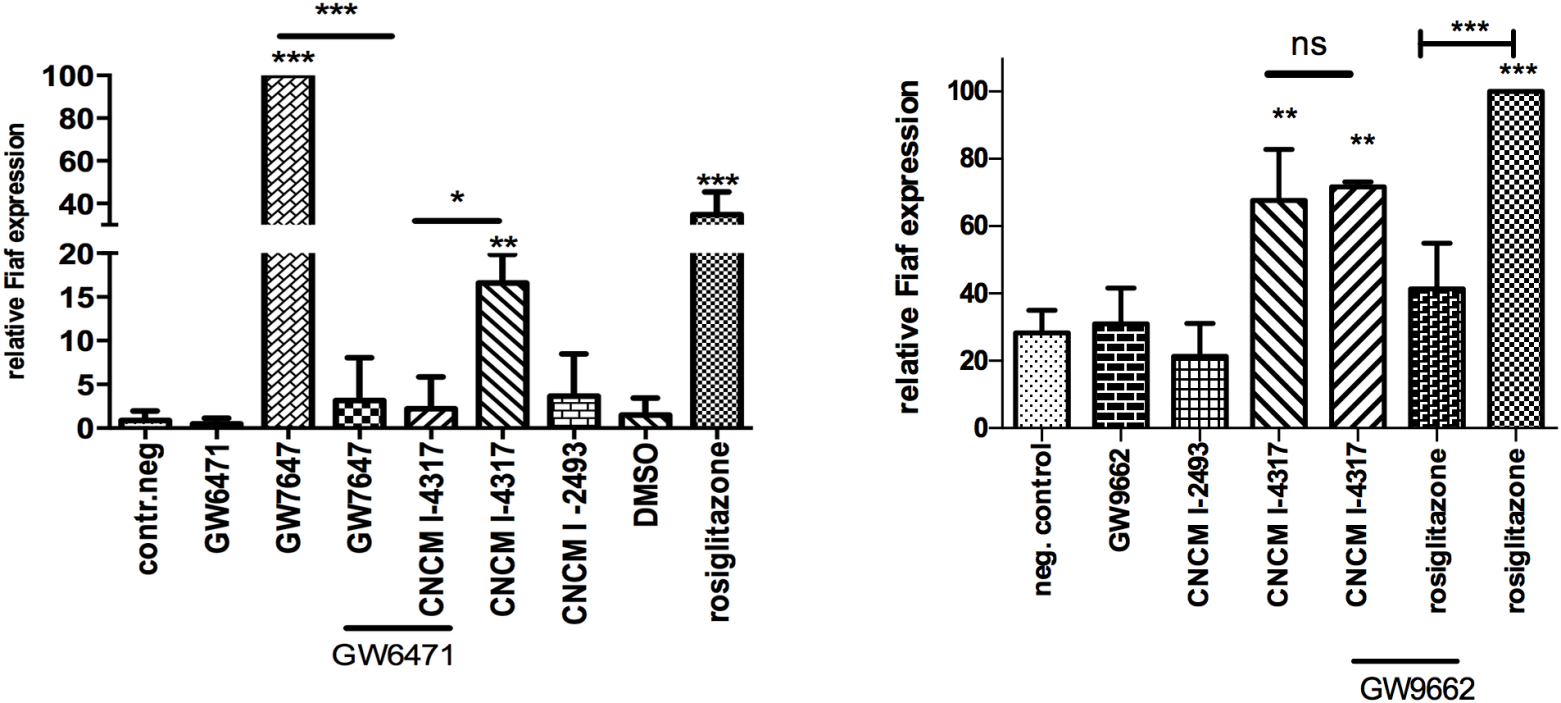
*L*. *rhamnosus* CNCMI–4317 may induce *F*iaf in a PPAR-α independent (a) but PPAR-γ dependent (b) manner. The antagonists (GW7647 and GW9662) were respectively added at 1 and 10μM 1h before challenging with agonists (GW6471 and rosiglitazone) during 6h. Bars represent means of *Fiaf* relative expression (percentage of rosiglitazone and GW7647 respectively) from three independent experiments performed in triplicates. Data are normalized using β-*Actin* as control gene and by GW7647 (a) or rosiglitazone (b). Stars represent p<0.05 (*), p< 0.01 (**) and p<0.001 (***) in comparison with negative control (DMEM). ns represent a non significant difference between *L*. *rhamnosus* CNCMI- 4317 versus *L*. *rhamnosus* CNCMI—4317 supplemented with GW9662.

Since *Fiaf* gene expression is controlled by both PPAR-γ and PPAR-α, we used specific ligands and inhibitors to investigate how *L*. *rhamnosus* CNCMI–4317 strain regulated *Fiaf* expression. Rosiglitazone and GW7647, agonists of PPAR-γ and PPAR-α respectively, increased the expression of *Fiaf* (*P*<0.001; Fig 2), whereas GW9662 and GW6471, the antagonists of PPAR-γ and PPAR-α respectively, strongly inhibited *Fiaf* gene expression (*P*<0.001). Additionally, *L*. *rhamnosus* CNCMI–4317 significantly induced *Fiaf* expression, which was completely abolished in the presence of GW6471 (*P*<0.05), indicating a PPAR-α dependent activation (Fig 2a). On the contrary, GW9662 did not modify *Fiaf* expression induced by the bacterial strain, suggesting that its effect was PPAR-γ independent (Fig 2b).

It is noteworthy that activation of PPAR-α resulted in a stronger regulation of *Fiaf* than PPAR-γ in our cellular model.

### *L*. *rhamnosus* CNCMI–4317 strain might act *via* a surface exposed molecule in IECs

**Fig 3:**
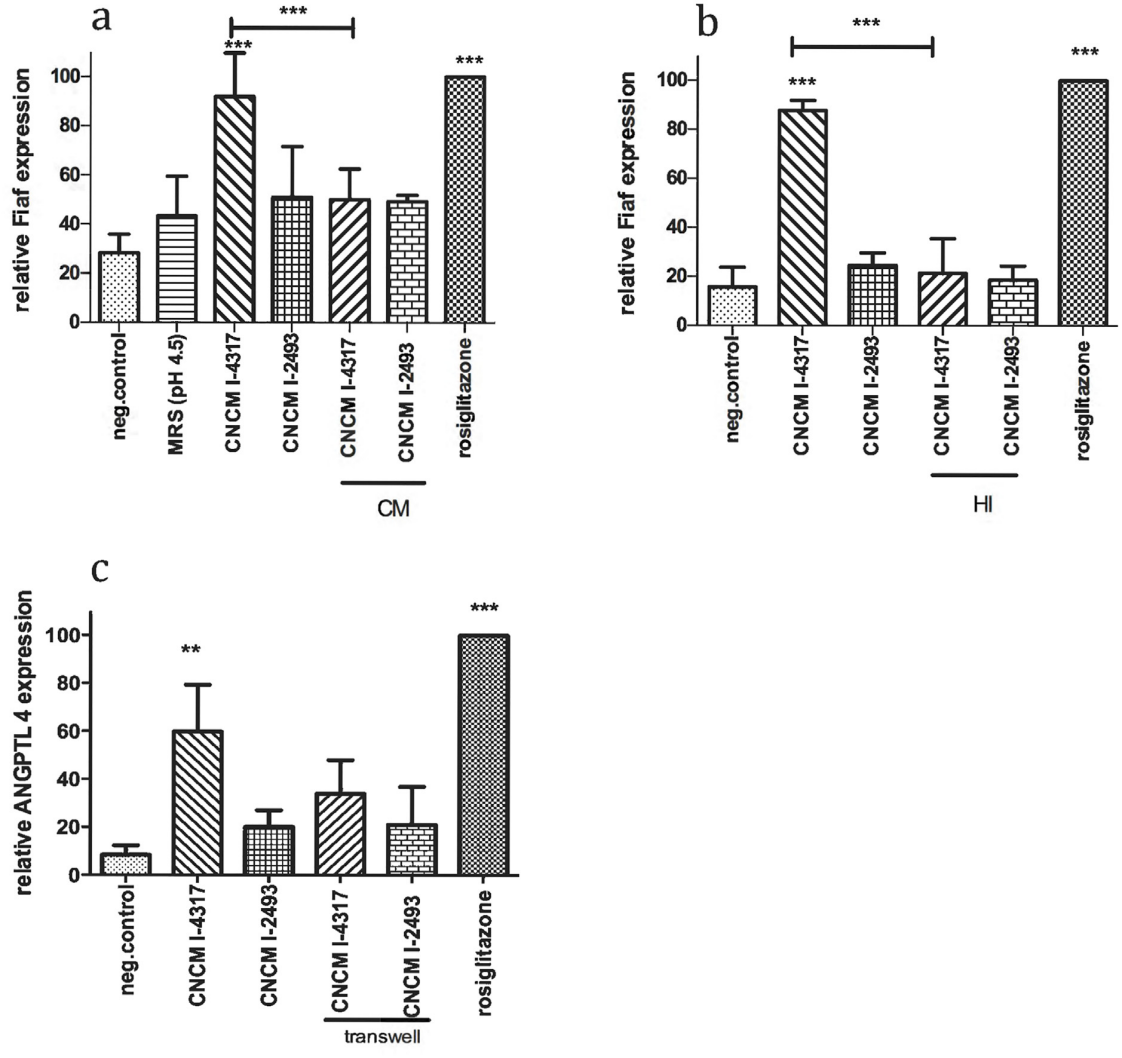
Bacterial effectors characterization. (a) Conditioned media, (b) Heat inactivated bacteria, (c) Transwell. Cells were incubated 6h with 10% of final volume of bacterial fractions (CM, HI) and 20% of bacteria for transwell structure. Transwell prevented contact between cells and bacteria. Bars represent means of *Fiaf* relative expression (percentage of rosiglitazone) from three independent experiments performed in triplicates. Data are normalized using β-*Actin* as control gene. Stars represent p<0.05 (*), p<0.01 (**) and p<0.001 (***) in comparison with negative control (DMEM). ns represents a no significant difference in comparison with negative control.

To determine the bacterial effector(s) involved in the activation of *Fiaf by* CNCMI–4317 strain, several bacterial fractions were tested. Conditioned medium (CM) (Fig 3a) and heat inactivated (HI) bacteria (Fig 3b) were not effective on *Fiaf* up-regulation suggesting that the effector was not a secreted product and was heat sensitive (*Fiaf* relative expression was: 50±12.53 and 21.33±14.22 respectively for CM and HI vs 92±17.69 and 87.67±4.16 for bacterial strain; *P*<0.001). The requirement of bacterial-cells direct contact was further assessed. To do so, HT–29 cells were separated from the bacteria using transwell^TM^ permeable support (Corning) in which the bacteria were added (Fig 3c). Using this item, the ability of the lactobacilli to induce *Fiaf* expression was reduced (33.98±14.03 vs 59.78±19.54) suggesting, at least in part, the requirement of a direct contact of bacterial surface factors with HT–29 cells.

### *L*. *rhamnosus* CNCMI–4317 modulated gene expression, cell death and survival, cellular growth and proliferation, immune response and lipid metabolism in IECs

**Fig 4:**
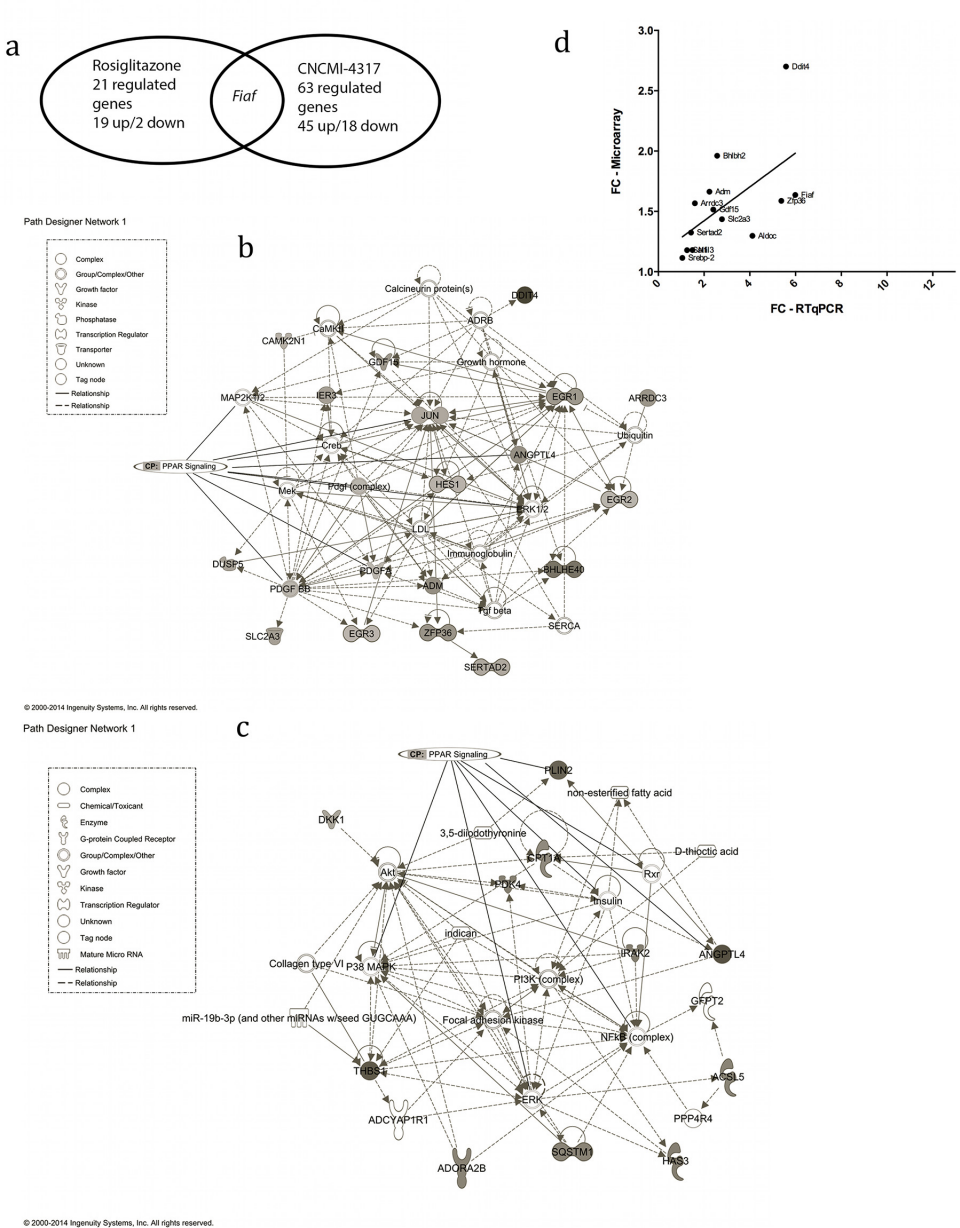
IECs transcriptome analysis in presence of *L*. *rhamnosus* CNCMI–4317 and rosiglitazone; (a) Venn diagram, (b) IPA networks detected when comparing *L*. *rhamnosus* CNCMI–4317 to negative control or rosiglitazone treatment (c) to negative control in IECs, (d) validation of microarray modulated genes by RT-qPCR. (b) FC are expressed in comparison with negative control (DMEM treatment), ns means that gene was not statistically significantly regulated by the treatment. Up-regulated genes are represented in grey shade except DKK1, which is down-regulated. (c) The networks included genes involved in neurological disease, cell cycle and cell development or Energy production, Lipid metabolism and small molecule biochemistry presented a score of 41 and 28 respectively (few genes are deleted to network for better view). The network displayed graphically as nodes (gene/gene products) and edges (the biological relationship between nodes). The node grey intensity indicates the expression of genes: black and bold: up-regulated, grey: down-regulated in intestinal tissues. The shapes of nodes indicate the functional class of the gene product. The log fold change values are indicated under each node. PPAR signalling canonical pathway was added. CP mean canonical pathway. (d) RT-qPCR data are normalized using geometrical mean of *β-Actin* and *Gapdh* as control genes.

We performed a whole genome transcriptome analysis of IECs in response to bacterial strains. HT–29 cells were incubated for 6 hours either with the bacterial strain of interest (*L*. *rhamnosus* CNCMI–4317), a control bacterium that did not induce *Fiaf* gene expression (*L*. *rhamnosus* CNCMI–2493), a culture medium as negative control, or rosiglitazone. We performed eight independent cultures of HT–29 cells at different passage number for *L*. *rhamnosus* CNCMI–4317, negative and rosiglitazone controls and four replicates for *L*. *rhamnosus* CNCMI–2493. In view of the strong effect of the cell culture (S1 Fig), we decided to include it as a covariable in the statistical model. We failed to detect genes significantly differentially expressed (DE) between *L*. *rhamnosus* CNCMI–2493 and the negative control (without bacterial strain), and we also hardly detected significant differences between the two bacteria *L*. *rhamnosus* CNCMI–4317 and CNCMI–2493 (data not shown). However, when comparing *L*. *rhamnosus* CNCMI–4317 strain and rosiglitazone to the negative control, respectively 63 and 21 genes were modulated (*P*<0.05). An Euler diagram visualization approach of these results highlighted that only *Fiaf* gene was commonly expressed (Fig 4a), strongly supporting the hypothesis that bacterial strain CNCMI–4317 acted in a PPAR-γ independent manner. As presented in Table 1, the most activated genes by *L*. *rhamnosus* CNCMI–4317 were *Ddit4* (DNA damage inducible transcript 4, fold change (FC) = 2.70, *qvalue* = 0.003), *Bhlbh2* (Basic helix loop helix family member 40, FC = 1.96, *qvalue* = 0.0005), *Adm* (Adrenomedullin, FC = 1.66, *qvalue* = 0.025) and *Fiaf* (FC = 1.63, *qvalue*<0.00089). To explore the molecular functions modified in response to *L*. *rhamnosus* CNCMI–4317, we measured the subsets of DE genes between treatments by using the core analysis function included in IPA software. Most biological functions found to be significantly enriched (*P*<0.05), by *L*. *rhamnosus* CNCMI–4317 were related to gene expression machinery, cell death/survival, cellular growth/proliferation, cell-mediated immune response and lipid metabolism categories (Table 1). Interestingly, those functions included canonical pathways associated with PPAR signalling, and HIF1α signalling (*P*<0.05) (S2 Fig). Four networks were identified with scores ranging from 41 to 19. The *Fiaf* gene was found to play a role in the regulatory network involved in putative functions such as neurological disease, cell cycle and cell development (Fig 4b). On the contrary, most of the genes regulated by rosiglitazone were involved in lipid or carbohydrate metabolism functions (Table 1). In this context, it is no surprisingly that the *Fiaf* gene was found to play a role in the regulatory network involved in energy production and lipid metabolism putative functions (Fig 4c). To validate technically the microarray gene expression data, IECs RNA in response to *L*. *rhamnosus* CNCMI–4317 were analysed by RT-qPCR for 12 genes (Table B in S1 File). RT-qPCR results confirmed the microarray expression levels with most genes having high r^2^ values (Fig 4d).

**Table 1:** List of regulated genes revealed by transcriptomic analysis after rosiglitazone or *L*. *rhamnosus* treatment of HT–29 cells.

Description		Adj p-value	FC rosiglitazone	FC CNCMI–4317
***Lipid metabolism (lipolysis)***				
*Angptl4/Fiaf*	Angiopoietin like 4	1,8.10^−4^/8,9.10^−4^	1,67	1,63
*Sertad2*	Serta domain containing 2	2,6.10^−2^	ns	1,32
*Vgf*	VGF nerve growth factor inducible	3,8.10^−2^	ns	1,14
*Ascl5*	Acyl-COA synthetase long-chain family member 5	2,9.10^−2^	1,19	ns
*Plin2*	Perilipin 2	3,2.10^−4^	1,51	ns
*Thbs1*	Thrombospondin 1	3.2.10^−4^	1,59	ns
*Cpt1a*	Carnitine palmitoyltransferase 1A (liver)	4,8.10^−2^	1,12	ns
*Elovl6*	ELOVL fatty acid elongase 6	3,8.10^−2^	1,31	ns
*Pex13*	Peroximal biogenesis factor 13	1,8.10^−2^	1,11	ns
***Carbohydrate metabolism***				
*Pdk4*	Pyruvate dehydrogenase kinase, isoenzyme 4	4.10^−2^	1,24	ns
*Sqstm1*	Sequestosome 1	3,3.10^−2^	1,19	ns
*Krt6a*	Keratin 6A	1,7.10^−2^	1,14	ns
*Has3*	Hyaluronan synthase 3	1,8.10^−2^	1,24	ns
*Scl2a3*	Solute carrier family 2 (facilitated glucose transporter), member 3	1,8.10^−2^	ns	1,43
*Stbd1*	Starch binding domain 1	3,8.10^−2^	ns	1,17
***Gene expression***				
*Zc3h8*	Zinc finger CCCH-type containing 8	3,8.10^−2^	ns	-1,18
*Axin2*	Axis inhibition protein 2	3,6.10^−2^	ns	-1,24
*Kctd11*	Potassium channel tetramerization domain containing 11	2,1.10^−2^	ns	1,33
*Sap30*	Sins 3A-associated protein, 30kDa	4,4.10^−3^	ns	1,26
*Ncoa5*	Nuclear receptor coactivator 5	2,1.10^−2^	ns	-1,29
*Mterf*	Mitochondrial transcription termination factor	3,8.10^−2^	ns	-1,14
*Egr3*	Early growth response 3	5,7.10^−3^	ns	1,16
*Hes1*	HES family bHLH transcription factor 1	3,8.10^−2^	ns	1,17
*Egr2*	Early growth response 2	1,17.10^−2^	ns	1,13
*Bhlbh2*	Basic-helix-loop-helix family, member 40	5,1.10^−4^	ns	1,96
*Egr1*	Early growth response 1	2,1.10^−2^	ns	1,51
*Eif5*	Eukaryotic translation initiation factor 5	3,4.10^−2^	ns	-1,13
***Cellular death and survival***				
*Adm*	Adrenomedullin	2,5.10^−2^	ns	1,66
*Rbm5*	RNA binding motif protein 5	2,3.10^−2^	ns	-1,27
*Ier3*	Immediate early response 3	4,9.10^−3^	ns	1,44
*Moap1*	Modulator apoptosis 1	1,6.10^−2^	ns	1,18
*Pim1*	Pim–1 oncogene	5,1.10^−4^	ns	1,47
*Pdrg1*	P53 and DNA damage regulated 1	2,9.10^−2^	ns	-1,23
*Uhrf1*	Ubiquitin-like with PDH and ring finger domain 1	3,3.10^−2^	ns	-1,15
*Dkk1*	Diskkopf WNT signaling pathway inhibitor 1	4,8.10^−2^	-1,16	ns
*Id2*	DNA binding 2, dominant negative helix-loop-helix protein	1,4.10^−2^	-1,15	ns
*Irak2*	Interleukin–1 receptor associated kinase 2	4.10^−2^	1,26	ns
***Cell-mediated immune response***				
*Jun*	Jun proto-oncogene	1,7.10^−2^	ns	1,33
*Ano6*	Anoctamin 6	2,6.10^−2^	ns	1,17
*Klf9*	Kruppel-like factor 9	3,8.10^−2^	ns	1,2
***Cellular growth and proliferation***				
*Ccnf*	Cyclin F	2,1.10^−2^	ns	-1,25
*Arrdc3*	Arrestin domain containing 3	2,8.10^−4^	ns	1,56
*Tubb2c*	Tubulin, beta 4B class IVb	3,3.10^−2^	ns	-1,3
*Camk2n1*	Calcium/calmodullin-dependent protein kinase II inhibitor	2,2.10^−2^	ns	1,21
*Gdf15*	Growth differentiation factor 15	4,4.10^−3^	ns	1,51
*Clk1*	CDC-like kinase 1	1,9.10^−2^	ns	1,2
*Dusp5*	Dual specific phosphatase 5	2,8.10^−2^	ns	1,39
*Zfp36*	ZFP36 Ring finger protein	2,1.10^−2^	ns	1,58
*Ddit4*	DNA-damage-inducible transcript 4	3,0.10^−3^	ns	2,7
*Pdgfa*	Platelet-derived growth factor alpha polypeptide	3,8.10^−2^	ns	1,14
*Ero1l*	ERO-like (*S*.*cerevisae*)	1,1.10^−2^	ns	1,2
***Other***				
*Mpzl2*	myelin protein zero-like 2	4,1.10^−2^	ns	1,1
*Trim8*	Tripartite motif containing 8	3,6.10^−2^	ns	1,15
*Foxd1*	Forkhead box D1	4,5.10^−2^	ns	1,17
*Ifrd1*	Interferon-related developmental regulator 1	3,4.10^−2^	ns	-1,21
*Axud1*	Cysteine-serine-rich nuclear protein 1	1,1.10^−2^	ns	1,32
*Gins3*	GINS complex subunit 3	3,3.10^−2^	ns	-1,21
*Iffo1*	Intermediate filament family orphan 1	4,9.10^−2^	ns	1,17
*Lmtk3*	Lemur tyrosine kinase 3	3,1.10^−2^	ns	1,32
*Heca*	Headcase homolog (Drosphila)	2,1.10^−2^	ns	1,34
*Rsrc2*	Arginine/serine coiled-coil 2	2,2.10^−2^	ns	1,2
*Znf689*	Zinc finger protein 689	3,3.10^−2^	ns	-1,17
*Oraov1*	Oral cancer overexpressed 1	3,6.10^−2^	ns	-1,23
*Ensa*	Endosulfine alpha	3,6.10^−2^	ns	1,19
*Slc39a10*	Solute carrier family 39 (zinc transporter), member 10	3,4.10^−2^	ns	1,12
*Ankrd37*	Ankyrin repeat domain 37	4,5.10^−3^	ns	1,7
*C1orf63*	Arginine/serine-rich protein 1	4,7.10^−2^	ns	1,14
*C7orf52*	Chromosome 7 open reading frame 52	2,1.10^−2^	ns	1,14
*C12orf47*	MAPKAPK5 antisense RNA 1	2,3.10^−2^	ns	1,27
*C1orf131*	Chromosome 1 open reading frame 131	3,3.10^−2^	ns	-1,29
*Irf2bp2*	Interferon regulatory factor 2 binding protein 2	1,1.10^−2^	ns	1,15
*C13orf34*	Bora aurora kinase A activator	4,1.10^−2^	ns	-1,24
*C7orf68*	Hypoxia inducible lipid droplet-associated	2,1.10^−2^	ns	1,38
*Frat2*	Frequently rearranged in advanced T-cell lymphomas 2	3,8.10^−2^	ns	1,15
*Adora2b*	Adenosine A2b receptor	4,1.10^−2^	1,2	ns
*Wdr37*	WD repeat domain 37	2,1.10^−2^	ns	-1,14
*Loc100132715*	Serine/arginine rich splicing factor 3 pseudogene	2.10^−2^	ns	-1,16
*Dsc2*	Desmocollin 2	1,3.10^−2^	1,2	ns
*Rhof*	Ras homologue family member F (in Filopodia)	1,3.10^−2^	1,23	ns
*Krt80*	Keratin 80	4,1.10^−2^	1,21	ns
*Tmem139*	Transmembrane protein 139	4.10^−2^	1,19	ns
*Ralgps2*	Ral GEF with PH domain and SH3 binding motif 2	4,1.10^−2^	1,17	ns
*Loc650832*	Similar to mitogen-activated protein kinase kinase 3 isoform A	4,8.10^−2^	1,23	ns

Fold change (FC) are expressed in comparison with negative control (DMEM treatment), ns means that gene was not statistically significantly regulated by the treatment

**Fig 5:**
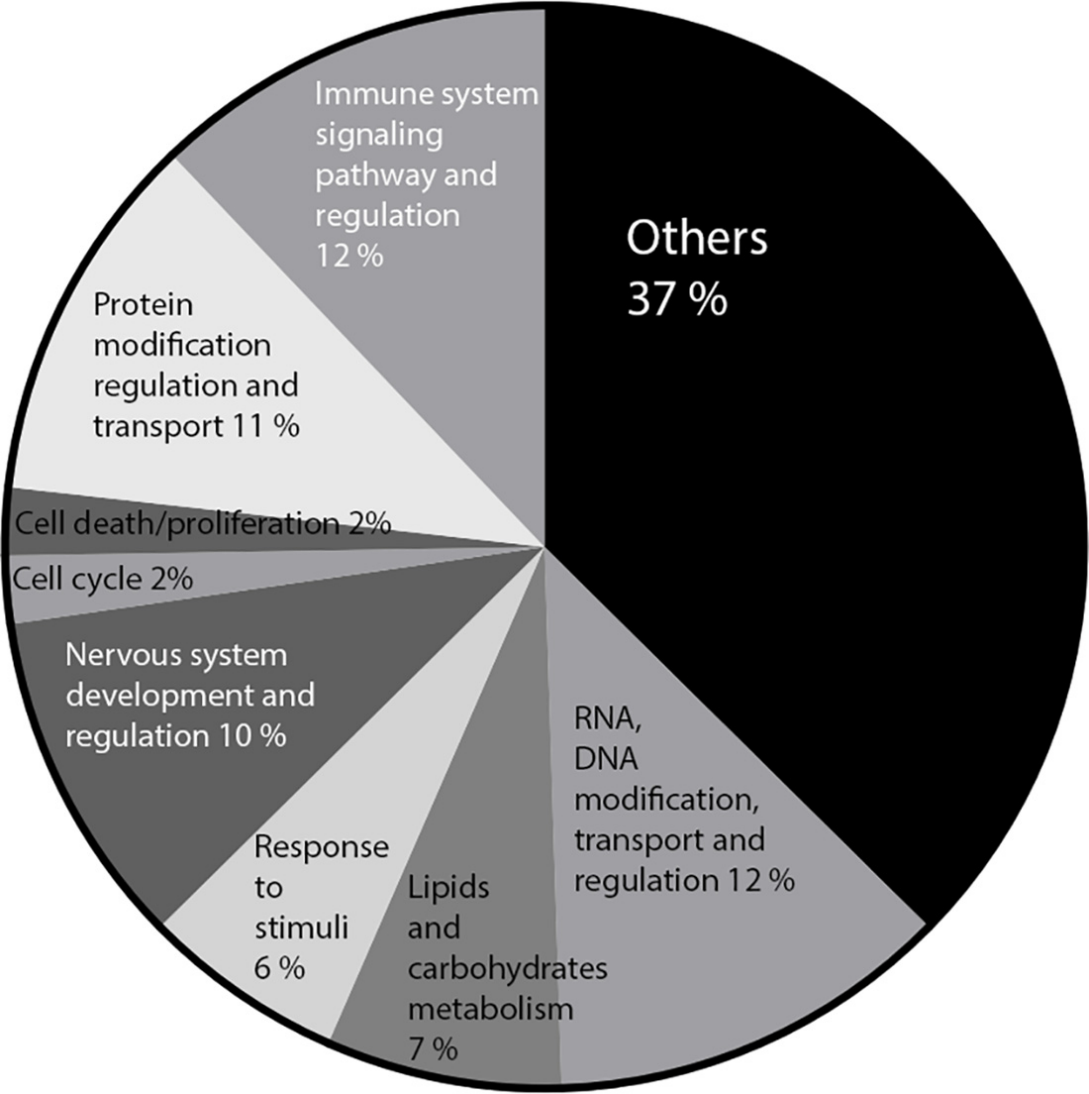
ErmineJ significant GO pathways modulated by *L*. *rhamnosus* CNCMI–4317 in IECs.

For physiological relevance, microarray data was also analysed at the level of gene sets that together encoded for particular differentially expressed functional GO terms by ErmineJ software (Fig 5). Notably, this analysis revealed that the gene sets involved in immune system signalling pathways and regulation or lipid and carbohydrate metabolism GO terms were enriched by *L*. *rhamnosus* CNCMI–4317 (*P*<0.05). Among the metabolic GO pathways regulated by our bacterial strain, 7 were shared with those induced by rosiglitazone treatment (data not shown).

### *In vivo* mono-colonization of Germ-free mice with *L*. *rhamnosus* CNCMI- 4317 increased plasma IL–7 and FIAF and tend to modulate *Fiaf* gene expression in the intestine

**Fig 6:**
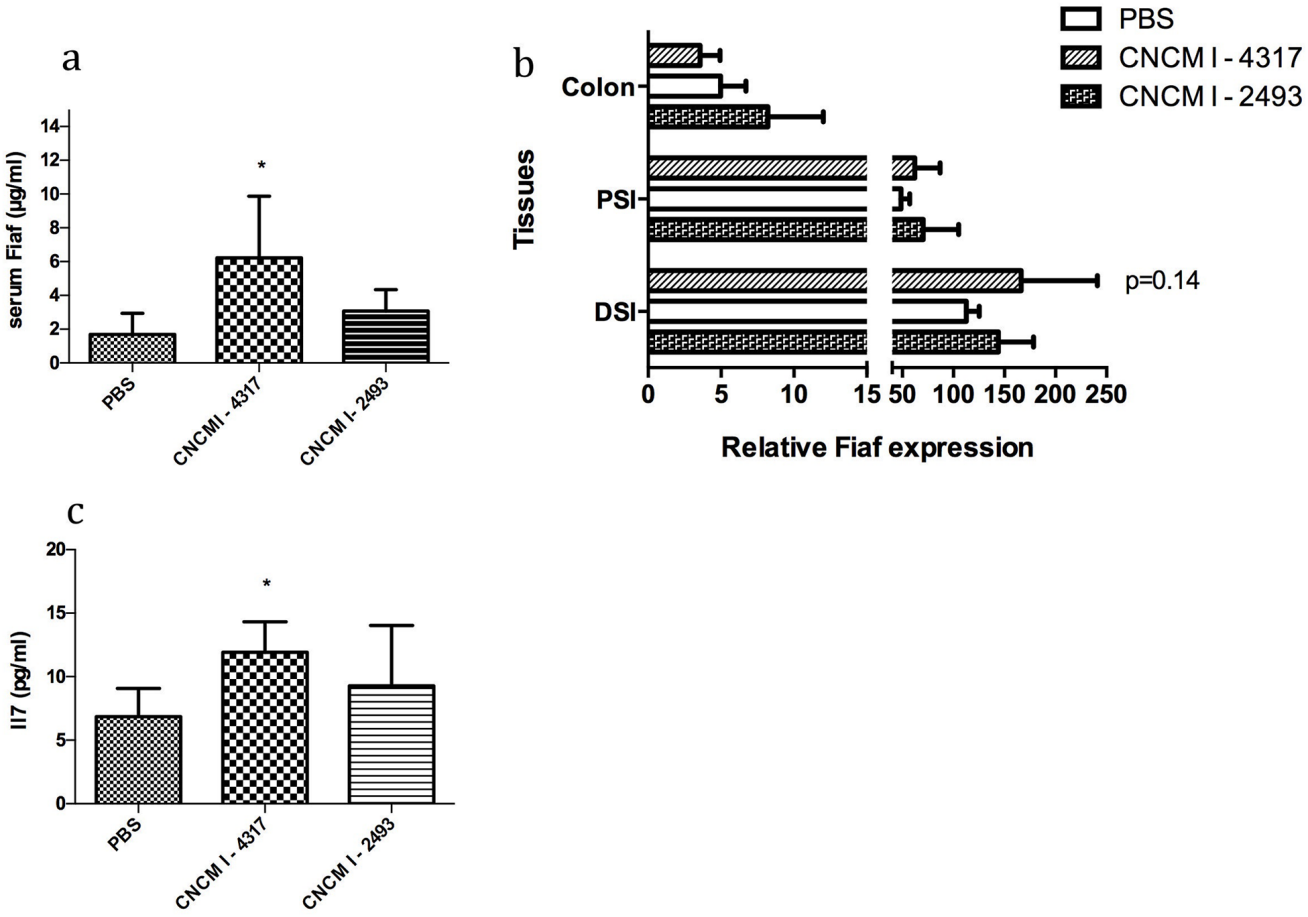
*In vivo*, (a) FIAF circulating level, (b) *Fiaf* expression in the gut, and (c) IL7. (a-c) Circulating *Fiaf* and Il–7 was measured using Elisa tests. (b) *Fiaf* expression was determined *via* RT-qPCR. Stars indicate p<0.05 (*) in comparison with GF control group (PBS). DSI (distal small intestine), PSI (proximal small intestine), P = 0.14 (Student’s t-test) corresponds to *L*. *rhamnosus* CNCMI– 4317 versus control (PBS).

Germ-free mice were colonized with *L*. *rhamnosus* CNCMI–4317 strain, *L*. *rhamnosus* CNCMI–2493 (control strain) or PBS during 11 days and then sacrificed. Mice colonized with *L*. *rhamnosus* CNCMI–4317 presented an increase in the concentration of plasma FIAF as compared to control mice (Fig 6a). With regard to the *Fiaf* gen*e* expression among different tissues, the *Fiaf* gene expression tended to increase in the distal small intestine in the presence of *L*. *rhamnosus* CNCMI–4317 (*P* = 0.14), but no significant differences could be observed for colonic expression (Fig 6b). Furthermore, circulating FIAF level was not correlated to the expression of *Fiaf* gene expression in adipose tissues nor in the liver (S3a and S3b Fig).

Cytokine levels in the serum of mice mono-colonized with bacterial strains were investigated. Only IL–7 was significantly higher (*P*<0.05) when comparing animals colonized with *L*. *rhamnosus* CNCMI–4317 strain versus GF mice or mice colonized with the control strain (Fig 6c). The other 8 cytokines were not significantly modified by the colonization (S3c Fig) and IFN-γ, IL–2, IL–4 and IL–6 were not detectable (data not shown).

Finally, FIAF circulating level is not correlated to a body weight gain (S3d Fig) nor lipoproteins level (S3e Fig) modifications.

## Discussion

It is now well established that the human gut microbiota is composed of 10^14^ bacteria and so represents a dynamic organ. Several bacteria are reported to play a role in host energetic metabolism regulation [17, 21, 24]. Several lactobacilli isolated from the human gut are widely used in dairy products. Assessment of their involvement in energy intake and storage appears crucial in the period when obesity is continuously growing worldwide. FIAF or ANGPTL4 has been identified as a key metabolism regulator. Intestinal epithelial cells (IECs) being the first line of contact between bacteria and the host represent an important interface for host physiology regulation by microbiota. In this context, we identified *L*. *rhamnosus* CNCMI–4317 strain as able to up regulate *Fiaf* gene expression in IECs. In order to identify the bacterial effector(s) responsible for modulating *Fiaf* expression, we tested several bacterial fractions. We showed that *Fiaf* regulation was not caused by a secreted compound and required the presence of live bacterial cells. Since the effect was abrogated by heat treatment, we hypothesized that surface exposed protein could be involved. Several beneficial metabolic effects have been reported under *Lactobacilli* treatment. These effects were linked to secreted compound as conjugated linoleic acids from *L*. *rhamnosus* P60 and *L*. *plantarum* P62 [24, 25] or unknown molecule [21]. One study mentioned the requirement of live *L*. *rhamnosus* GG cells to decrease serum glucose levels in a diabetic mice model [26]. However, our work showed for the first time that *L*. *rhamnosus* CNCMI–4317 strain could play a role on host metabolism through the regulation of *Fiaf* expression in the epithelial cell *via* a direct contact.

PPARs isotypes play an important role in *Fiaf* regulation [14, 27, 28]. A recent study provided evidence that *L*. *paracasei* F19 upregulates *Fiaf* expression in IECs in a PPAR-α and PPAR-γ dependent manner [21]. In our study, tested *L*. *paracasei* strains did not regulate *Fiaf* expression, highlighting a strain specific effect, which was also seen for our *L*. *rhamnosus* strain. Our results suggest a PPAR-α dependency, but rule out a role for PPAR-γ. On the contrary to a recent study from Alex *et al* (2013), and in agreement with Aronssson *et al* (2010), ours experiments showed that PPAR-α regulates *Fiaf* and even induced a stronger activity than PPAR-γ in HT–29 cells. In order to determine the mechanism of action involved, we performed a whole genome transcriptome analysis of IECs in contact with different bacterial strains. We detected a strong effect of independent cell culture passage, driving us to include it as a covariable in our statistical model. Unfortunately, the low number of replicates probably unabled us to identify genes differently regulated. However, a total of 63 annotated genes were revealed as significantly different between *L*. *rhamnosus* CNCMI–4317 and negative control. The IPA analysis of these genes disclosed that they encoded for molecular functions involved in PPAR and HIF1 pathways. In agreement, published data provide evidences for *Fiaf* regulation by PPAR and hypoxia [29]. However, the absence of effect of conditioned media excluded two known major potential regulators, namely H_2_O_2_ [18] and SCFA [19].

Interestingly, genes affected by *L*. *rhamnosus* CNCMI–4317 were mainly involved in cellular growth/proliferation, cell death, immune response and lipid metabolism. In agreement with our findings, others strains of *L*. *rhamnosus* have been described as cellular growth and proliferation modulators *in vivo* suggesting potent lactobacilli shared effect [30, 31]. Moreover, *L*. *rhamnosus* CNCMI–4317 regulated several transcription factors involved in gene expression and neurological diseases, cell cycle and cellular development. In this context, *Fiaf* did not appear in the network of energy production and lipid metabolism as rosiglitazone confirming a different mechanism of action between both treatments. However, few unregulated but intermediate genes in the neurological diseases, cell cycle and cellular development network were correlated to metabolism (*Ldl*, *Erk1/2*, *Map2k1/2*, *Creb*, *Mek*) especially through PPAR pathway. This underlines a potent role of *L*. *rhamnosus* CNCMI–4317 strain in host metabolism as revealed by the regulation of expression of *Scl2a3* (solute carrier family 2, member 3) and *Gdf15* (growth differentiation factor 15). The last, known for its role in cellular cycle has been recently involved in T2D [32, 33]. Despite an evident *in vitro* regulation of *Fiaf* leading by PPAR-α, transcriptome analysis exhibited that the majority of genes were not regulated by PPAR-α. These results suggest that our bacterial strain could modulate multiple cellular functions by complex and diverse mechanisms.

In order to validate *in vivo* the cellular regulation of *Fiaf* observed *in vitro*, we colonized C57BL/6 mice with *L*. *rhamnosus* CNCMI–4317. We observed a higher level of circulating FIAF and an increased tendency expression of *Fiaf* gene in the small intestine, although non-statistically significant. However, *Fiaf* was not regulated in the colon. These data correlate with Korecka *et al* (2013), who showed different *Fiaf* level expression in gastrointestinal (GI) tract under bacterial administration due to different microbial population and fermentation in conventional model [19]. In our case, it may be explained by Lactobacilli colonization in GI upper part in GF model and absence of SCFA release (potent *Fiaf* activator) in colon. Additionally, no modulation of *Fiaf* gene expression in liver or adipose tissues was observed upon colonization with *L*. *rhamnosus* CNCMI–4317 strain. Neither serum lipoproteins level nor body weight was affected in comparison with control GF mice. Taken together, our data suggest that an up-regulation of circulating *Fiaf* was not associated with lipoproteins levels. This is in disagreement with Aronsson *et al* (2010), who showed a correlation between plasma FIAF and VLDL TGs levels in mono-colonized mice [21]. However, Grootaert *et al* (2011) suggested an importance of FIAF isoform in specific physiological effect [18]. Thus, the discrepancies with our results may come from technical differences (Western blot *vs* Elisa) targeting different isoforms.

Furthermore, *L*. *rhamnosus* CNCMI–4317 strain induced serum IL–7 suggesting a role in immune cell development/regulation. This is in agreement with a recent *ex vivo* human transcriptome analysis showing the ability of *L*. *plantarum* strain to induce IL–7 in the duodenum and suggesting a potent common property of Lactobacilli strains [31].

Finally, our *in vivo* study failed to identify strong *Fiaf* regulation in different tissues and impact on host metabolism but we may expect that *Fiaf* exerts a higher physiological effect on more complex environment, for example in rodent model exhibiting enhanced metabolic profiles (i.e. high fat diet).

In the context where bacterial regulation of *Fiaf* appears to play a central role in fat storage, we provide evidences for the potential role of one particular *L*. *rhamnosus* strain as a *Fiaf* regulator *in vitro*. It is noteworthy that the effect is strain specific. To go deeper in the understanding of *Fiaf* involvement in host metabolism and to better understand strains specificities involved in this phenomenon, it will be important to study the impact of this bacterial strain on the physiology of conventional mice exposed to high fat diet and in human tissue set-up.

## Supporting Information

S1 Fig Assessment of microarrays variability by (a) multidimensional scaling analysis (MDS), (b) hierarchical clustering. (TIFF)

S2 Fig IPA canonical pathways when comparing *L*. *rhamnosus* CNCMI–4317 (empty bars) or rosiglitazone (hatched bars) to negative control. (TIFF)

S3 Fig (a) *Fiaf* expression levels in adipose tissue (b) and in liver, (c) cytokines (d) body weight, and (e) serum lipoproteins in C57BL/6 mice. (TIF)

S1 File Material and method in S1 file. Table A in S1 file: List of tested bacteria. Table B in S1 file: List of Taqman probes.
(DOCX)
