# Supplementary figures and images for: Lactobacillus rhamnosus CNCMI-4317 Modulates Fiaf/Angptl4 in Intestinal Epithelial Cells and Circulating Level in Mice
Source: PLoS One. 2015 Oct 6;10(10):e0138880. doi: 10.1371/journal.pone.0138880 (PMC4595210; doi:10.1371/journal.pone.0138880)

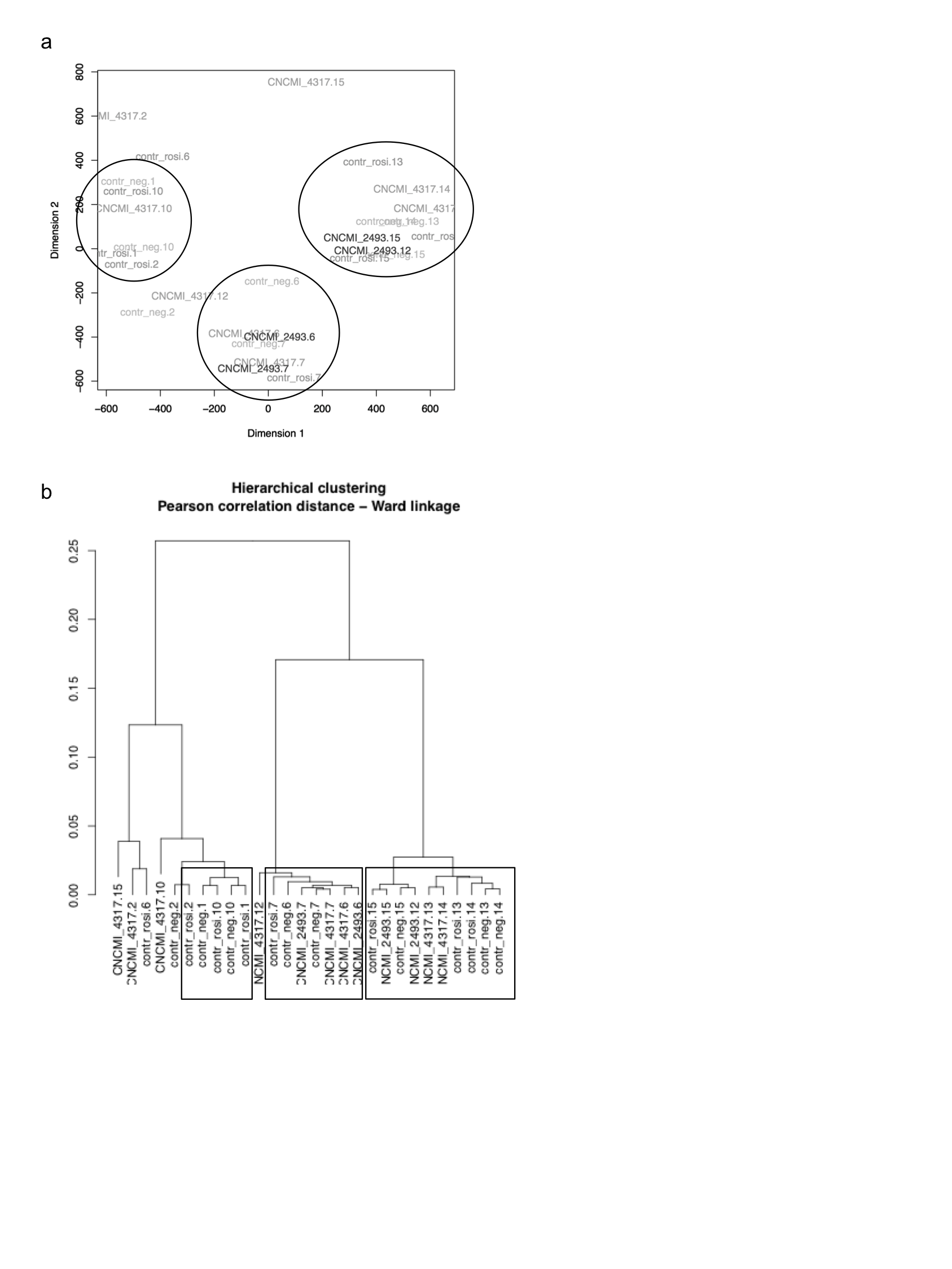

Supplement: S1 Fig — (TIFF) [file pone.0138880.s001.tiff]

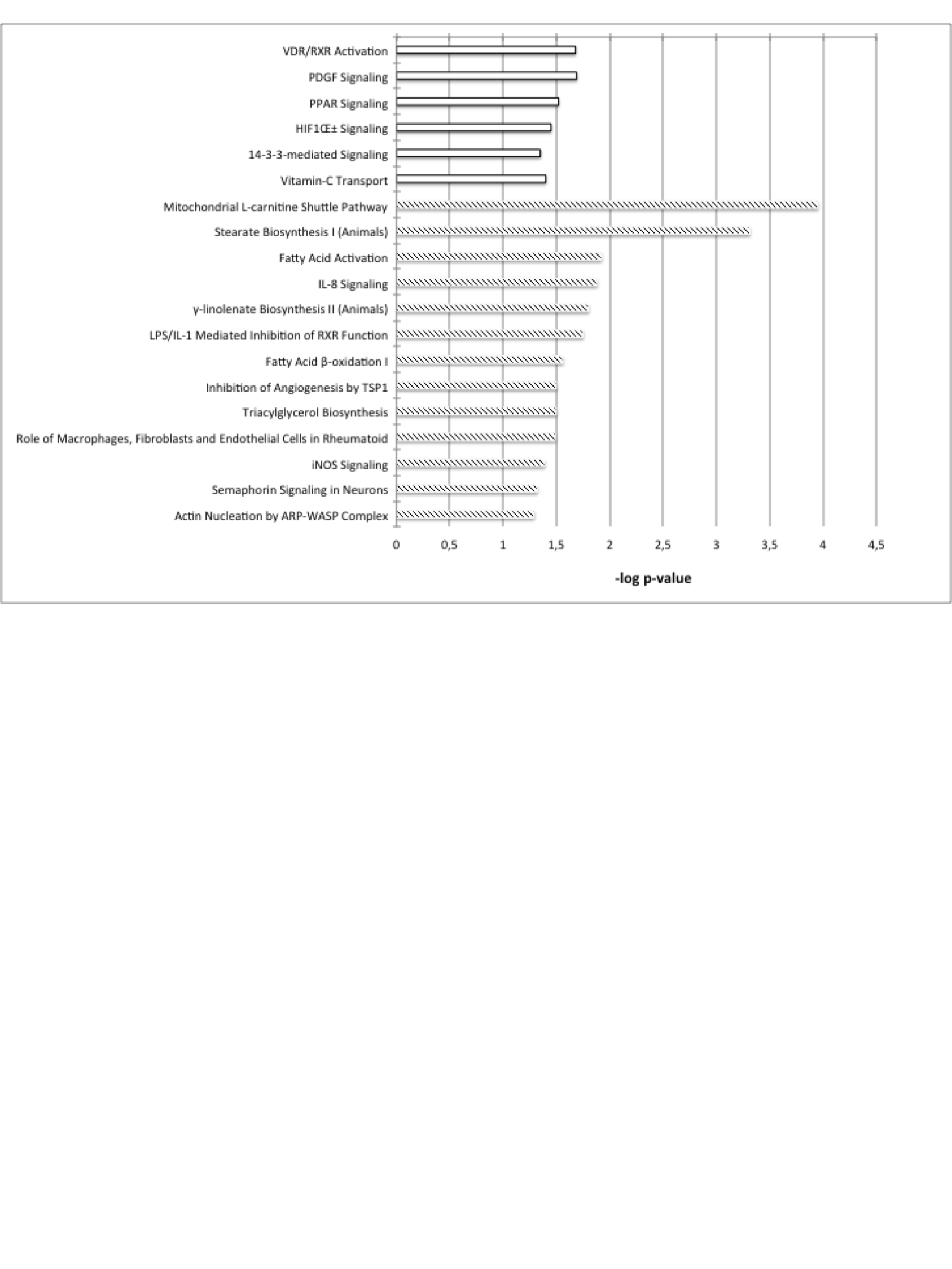

Supplement: S2 Fig — (TIFF) [file pone.0138880.s002.tiff]

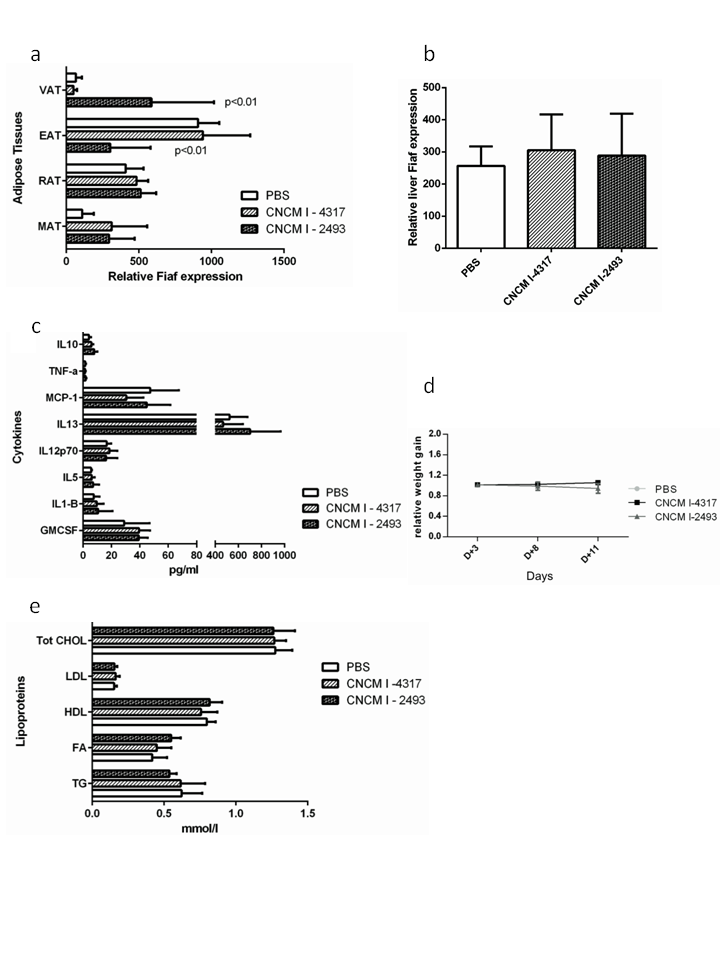

Supplement: S3 Fig — (TIF) [file pone.0138880.s003.tif]
