# Supplementary material for: Lactobacillus rhamnosus CNCMI-4317 Modulates Fiaf/Angptl4 in Intestinal Epithelial Cells and Circulating Level in Mice
Source: PLoS One. 2015 Oct 6;10(10):e0138880. doi: 10.1371/journal.pone.0138880 (PMC4595210; doi:10.1371/journal.pone.0138880)
Supplement: S1 File — Table A in S1 file: List of tested bacteria. Table B in S1 file: List of Taqman probes. (DOCX) [file pone.0138880.s004.docx]

### Supplementary data

Material and method

Microarray analysis of HT-29 cells

Data normalization: The homogeneity of the background was systematically checked on each microarray by the boxplot and image plot procedures of the linear models for microarray data ('Limma' library; version 3.16.8).

To avoid inclusion of genes with no expression, the minimum threshold of probe intensity was chosen based on an inter quartile range (IQR) offset of 0.25 for at least one array per treatment. This control filtering criteria reduced the number of genes from 34.694 to 26.020. In order to discover biologically important changes in expression, the robust spline normalization (RSN) of lumi package was applied. This function combines the features of quintile and loess normalization and is designed to normalize the variance-stabilized data. The multidimensional scaling plots (MDS), which measure the similarity of the samples and project it into two dimensions, were performed to measure the relationship between samples based on multidimensional scaling, through the 'FactoMineR' library (version 1.25).

The determination of differentially expressed genes using the linear models for microarray was performed ('Limma' package). The statistical model included the treatment type as fixed effect, and the cell culture passage as covariable. Treatment type was defined as (1) *L. rhamnosus* CNCMI-4317, (2) *L. rhamnosus* CNCMI-2493, (3) negative control and (4) control positive (rosiglitazone). Each of the treatment groups was compared using the contrast argument of the “makeContrasts” function of the Limma package. The *P*-values were corrected for multiple testing using a false discovery rate method (*q*-value < 0.05), which provides an estimate of the fraction of false discoveries among the significant terms.

Centred on significantly expressed genes, unsupervised analysis was done to visualize clusters of samples based on their variance-covariance structure. Such an analysis helps to define coordinated regulation of similarly related genes and study fundamental and intrinsic differences at the level of transcription that are specific to the groups studied. Thus, a two-way hierarchical cluster analysis was performed using 'hclust' function with '1-cor (x) ' as distance and 'ward' as aggregation criterion. The 'heatmap' function was used to generate images. In addition, PCA was performed with 'FactoMiner' library to better identify, which genes contribute most to the separation of expression patterns between groups.

Genes selection for RT-qPCR validation: The genes were selected based on the following strategies: 1- genes with significant differential expression levels between the phenotypes of interest that spanned a dynamic range of at least log2 (ratio) > 0.485; 2- genes with biological interest (e.g. *Aldoc*).

Table 1: List of tested bacteria.

| **Bacteria** | **Species** | **Origins** |
| --- | --- | --- |
| CNCMI-4316 | *L. rhamnosus* | Cheese |
| CNCMI-4317 | *L. rhamnosus* | Cheese |
| Lr9 | *L. rhamnosus* | Plant |
| Lr110 | *L. rhamnosus* | Animal gut |
| Lr108 | *L. rhamnosus* | Human gut |
| CNCMI-2493 | *L. rhamnosus* | Type strain |
| CNCMI-6390 | *L. rhamnosus* | Cheese |
| Lr64 | *L. rhamnosus* | Dairy product |
| Lr52 | *L. rhamnosus* | Endocarditis |
| CNCMI-3689 | *L. paracasei* | Dairy product |
| Lpp225 | *L. paracasei* | Animal gut |
| Lpp120 | *L. paracasei* | Dairy product |
| CNCMI-1518 | *L. paracasei* | Dairy product |
| Lpp226 | *L. paracasei* | Dairy product |
| Lpp49 | *L. paracasei* | Plant |
| Lpp22 | *L. paracasei* | Dairy product |
| Lpp223 | *L. paracasei* | Human feces |
| Lpp46 | *L. paracasei* | Plant |
| Lpp37 | *L. paracasei* | Dairy product |

**Table 2: List of Taqman probes.**

| **Probes** | **References** |
| --- | --- |
| Hs01060665_g1 | H *β-Actin* |
| Hs02758991_g1 | H *Gapdh* |
| Hs00171132_m1 | H *Gdf15* |
| Hs00705412_s1 | H *Nfil3* |
| Hs00902800_g1 | H *Aldoc* |
| Hs00207372_m1 | H *Sertad2* |
| Hs00185658_m1 | H *Zfp36* |
| Hs00161511_m1 | H *Sat1* |
| Hs01081784_m1 | H *Srebp2* |
| Hs00385845_m1 | H *Arrdc3* |
| Hs01111686_g1 | H *Ddit4* |
| Hs01041212_m1 | H *Bhlbh2* |
| Hs01101127_m1 | H *Angptl4* / *Fiaf* |
| Hs00969450_g1 | H *Adm* |
| Hs00359840_m1 | H *Slc2a3* |

Supplementary figures :

Supplementary Figure 1: Assessment of microarrays variability by (a) multidimensional scaling analysis (MDS), (b) hierarchical clustering. Treatments are indicated by different grey shade (black represent *L. rhamnosus* CNCMI-2493, dark; rosiglitazone, light grey; negative control, medium grey; *L. rhamnosus* CNCMI-4317 replicates). Circles take together trials with lower variability.

Supplementary Figure 2: IPA canonical pathways when comparing *L. rhamnosus* CNCMI–4317 (empty bars) or rosiglitazone (hatched bars) to negative control. Only canonical pathways that presented a −log P-values exceeding 1,30 (FDR q-values <0.05) were preserved.

Supplementary Figure 3: (a) *Fiaf* expression levels in adipose tissue (b) and in liver, (c) cytokines (d) body weight, and (e) serum lipoproteins in C57BL/6 mice. Body weight is normalized by D0 (First day of gavage), FA (Fatty acids), TG (tryglycerids), HDL (High density lipoprotein), LDL (Low density lipoprotein), Tot CHOL (Total cholesterol), VAT (visceral adipose tissu), EAT (epidydimal adipose tissue), RAT (retroperitoneal adipose tissue), MAT (mesenteric adipose tissue). IL-10 was detected for 3 GF mice and 2 mice, which received CNCMI-2493 only, compared to mice, which received *L. rhamnosus* CNCMI-4317 and which expressed IL-10.
